# Comparison of coronary arterial lumen dimensions on angiography and plaque characteristics on optical coherence tomography images and their changes induced by statin

**DOI:** 10.1186/s12880-016-0166-4

**Published:** 2016-11-22

**Authors:** Nana Dong, Zulong Xie, Wei Wang, Jiannan Dai, Meng Sun, Zhongyue Pu, Jinwei Tian, Bo Yu

**Affiliations:** 1 Department of Cardiology, 2nd Affiliated Hospital of Harbin Medical University, 246 Xuefu Road, Nangang District, Harbin, 150086 China; 2 Key Laboratory of Myocardial Ischemia, Harbin Medical University, Ministry of Education, 246 Xuefu Road, Nangang District, Harbin, 150086 China

**Keywords:** Optical coherence tomography, Coronary angiography, Atherosclerosis, Statin

## Abstract

**Background:**

Coronary angiography (CAG) is widely used to assess lumen dimensions, and optical coherence tomography (OCT) is used to evaluate the characteristics of atherosclerotic plaque. This study was aimed to compare coronary lumen dimensions using CAG and plaque characteristics using OCT and their changes during statin therapy.

**Methods:**

We identified 97 lipid-rich plaques from 69 statin-naïve patients, who received statin therapy in the following 12 months. CAG and OCT examinations were conducted at baseline and 12-month follow-up period.

**Results:**

Lesion length, as measured by CAG, was closely correlated with lipid length by OCT (baseline: *r* = 0.754, *p* < 0.001; follow-up: *r* = 0.639, *p* < 0.001). However, no significant correlations were found between the other findings on OCT and data on CAG. With 12-month statin therapy, microstructures of lipid-rich plaques were significantly improved, but CAG-derived lumen dimensions were not improved. Moreover, we found no significant relationship between changes in OCT measurements and changes in CAG data over time.

**Conclusion:**

Lipid length on OCT and lesion length on CAG were closely correlated. However, plaque microstructural characteristics on OCT showed no significantly statistically correlations with lumen dimensions on CAG, neither did their evolutionary changes induced by statin over time.

**A retrospectively registered study:**

Clinical trial registry: *ClinicalTrial.gov*. Registered number: NCT01023607. Registered 1 December 2009.

## Background

Patients with rupture-prone lesions in the coronary arteries, also known as “vulnerable plaques”, are at risk of acute coronary syndrome (ACS) [1, 2]. Morphological characteristics of vulnerable plaques tend to possess a large lipid core and a thin fibrous cap overlying inflammatory-cell infiltration [3, 4]. Statin-based lipid-modulating therapy has been documented to stabilize vulnerable plaque and reduce atheroma volume [5–7]. Catheter-based diagnostic techniques, such as coronary angiography (CAG), optical coherence tomography (OCT) and intravascular ultrasound (IVUS), provide more accurate histopathological characteristics of coronary lesions and are widely applied in clinical examination of coronary arteries before or after percutaneous coronary interventions and as adjunctive devices to assess lesion characteristics or lumen and stent situations in detail [8–10]. OCT, with a high resolution of approximately 10―20 μm, allows for the assessment of the microstructures in plaques and has recently been regarded as an “optical biopsy” imaging modality that can visualize the morphological characteristics and compositions of plaques in situ and in real time [4, 11–13]. It is generally known that CAG is widely used to assess lumen dimensions, and display the lumen as a longitudinal and cross-sectional silhouette [14–16]. The current study was aimed to identify the relationship between lumen dimensions as seen on CAG and plaque microstructures as seen on OCT, and the relationship between their changes during statin intervention.

## Methods

### Study population

This was a retrospective study of a single blind and randomized clinical trial registry (ClinicalTrial.gov registered number: NCT01023607), designed to assess the effects of statins on lipid-rich plaques. The inclusion criteria were as follows: 1) were statin-naïve (i.e., those who were not on statins) patients with one or more mild-to-moderate lesion(s); 2) had non-culprit lesions identified as lipid-rich plaques by OCT; 3) were to receive statin treatment during the following 12–month follow-up period; and 4) were to undergo CAG and three-vessel OCT imaging procedures during the 12–month follow-up period. The exclusion criteria were as follows: 1) a life expectancy ≤12 months; 2) contraindications to oral statins; 3) severe hepatic or renal dysfunction; or 4) congestive heart failure. Fifty-one patients including those who didn’t have the follow-up information (*n* = 31), had poor-quality images from OCTs (*n* = 13), and mismatched imaging (*n* = 7) were excluded. After screening, 69 patients (62.3% male; age: 55.7 ± 9.4 years) with 97 coronary non-culprit, lipid-rich plaques (30.1% located in the left anterior descending artery, 46.6% in right coronary artery, 17.5% in left circumflex artery) were analyzed in the current study. This study was approved by the institutional review board of the Second Hospital of Harbin Medical University (Harbin, China), and all patients provided written informed consent before any procedures were initiated.

### Angiographic and OCT images follow-up

According to the study protocol, follow-up angiography and three-vessel OCT images were performed at baseline and 12 months. Quantitative angiographic of the coronary arteries was performed to determine the minimal lumen diameter (MLD), diameter stenosis (DS) and lesion length as previously described in detail [17]. The interpreter was blinded to OCT findings. Analyses of the baseline and follow-up coronary angiograms were conducted using the Cardiovascular Angiography Analysis System (CAAS 5.10, Pie Medical Imaging B.V., Maastricht, the Netherlands).

After detecting by CAG and dealing with the culprit/target lesions, intracoronary OCT images were performed as previously described [4, 5]. In short, with the TD OCT system, a 3-F occlusion balloon catheter was advanced proximal to the lesion and inflated at 0.5 atm. to 0.7 atm. During image acquisition, lactate Ringer’s solution was infused intra-coronary from the distal tip of the occlusion balloon catheter at 0.5 ml/s to 2.0 ml/s by a high-pressure injector. The OCT wire was pulled back from a distal to a proximal position automatically at a rate of 1.0 mm/s. The fibrous-cap thickness (FCT) was measured 3 times at the thinnest place, and the values were averaged. A lipid-rich plaque was defined as previously that plaque with fibrous-cap thickness < 120 μm and lipid arc > 100° [5]. In lipid-rich plaque, the lipid arc was measured at 1 mm interval, the maximum value was recorded as maximum lipid arc, and the mean value was calculated as mean lipid arc. Lipid length was measured on a longitudinal view. Lipid index, was calculated as the mean lipid arc multiplied by lipid length [18]. A thin-cap fibroatheroma (TCFA) has rich lipid core and FCT < 65 μm, while a thick-cap fibroatheroma (ThCFA) was a LRP with a thick fibrous cap (≥65 μm) [3, 19]. Macrophage accumulation was characterized as increased signal intensity, combined with heterogeneous backward shadows within the lesion. Microvessel, also named neovascularization, appears as signal-free tubuloluminal structure with a diameter of 50–300 μm that was observed as ≥ 3 consecutive cross-sectional OCT images [20]. Beside lipid pool, the thin and linear region of high intensity was cholesterol crystal [21]. Intraluminal thrombus was identified as protruding masses attached to the arterial wall [22]. Calcification was recorded when an area showed low backscatter signal and a sharply delineated border. Plaque disruption was a discontinued fibrous membrane with underlying communication between the lumen and the cavity.

All OCT images were analyzed using proprietary offline software (LightLab Imaging, Inc., Westford, MA, USA) by two independent, experienced observers. In cases in which was disagreement between them, a third independent investigator helped reach a consensus on image matching and quality. All investigators of CAG and OCT images were blinded to the patient’s conditions and the study protocol, and all images were digitally stored for future analyses. In the follow up studies, repeated measurements from the same reference points were performed, and the measurements of CAG and OCT between the two points were determined using computer software.

### Statistical analysis

All statistical analysis was performed by an independent statistician using IBM SPSS version 17.0 (SPSS Inc., Chicago, Illinois, USA). Data are expressed as mean ± standard deviation (SD) or counts and proportions. Quantitative data at baseline and at follow-up are compared using paired *t* test and qualitative data using McNemar test. Continuous variables between TCFAs and ThCFAs were compared by using unpaired *t* test. The categorical variables were analyzed using the Chi-square or Fisher exact test. Pearson or Spearman ranks test was used to investigate the relationship between CAG findings and OCT measurements, and also the correlations between changes in CAG and changes in OCT measurements. A *p*-value < 0.05 was considered to be statistically significant.

## Results

The baseline clinical characteristics of the patients are shown in Table 1. As shown in Table 2, with statin treatment, the level of low-density lipoprotein cholesterol (LDL-C) was decreased by 25.4% (from 109.4 ± 24.6 mg/dL baseline to 73.9 ± 29.2 mg/dL follow-up, *p* < 0.001). In addition, except for high-density lipoprotein cholesterol (HDL-C), the total cholesterol, triglycerides, and apolipoprotein B were all markedly improved (*p* < 0.001, *p* < 0.001, *p* < 0.001, *p* = 0.005, respectively).

**Table 1:** Baseline clinical characteristics

Baseline characteristics	
Patients number	*n* = 69
Age, years	55.7 ± 9.4
Male	43 (62.3)
Hypertension	44 (63.8)
Diabetes	34 (49.3)
Smoking	33 (47.8)
Previous MI	15 (21.7)
Prior PCI	12 (17.4)
ACS	55 (79.7)
SAP	14 (20.3)
Concomitant drugs at follow-up:	
ACEI/ARB	31 (44.9)
Beta blocker	42 (60.9)
CCB	19 (27.5)
Aspirin	68 (98.6)
Clopidogrel	68 (98.6)
Nitrate	40 (60.0)
Angiographic Findings:	
Plaques numbers	*n* = 97
Left Anterior Descending	31 (30.1)
Right Coronary Artery	48 (46.6)
Left Circumflex	18 (17.5)

Data were presented as mean ± standard deviation (SD) or number (%) except for SD. *ACEI* angiotensin-converting enzyme inhibitor, *ACS* acute coronary syndrome, *ARB* angiotensin II receptor blocker, *CCB* calcium channel blocker, *SAP* stable angina pectoris, *Previous MI*, previous myocardial infarction, *Prior PCI* prior percutaneous coronary intervention

**Table 2:** Serum lipid results, CAG and OCT measurements at baseline and follow-up

Variables	Baseline	Follow-up	*p*-value
Serum lipid			
Total cholesterol, mg/dl	198.6 ± 42.2	146.1 ± 40.0	<0.001
LDL-C, mg/dl	109.4 ± 24.6	73.9 ± 29.2	<0.001
HDL-C, mg/dl	49.3 ± 12.3	46.7 ± 13.9	0.123
LDL-C/HDL-C	2.3 ± 0.8	1.7 ± 0.7	<0.001
Triglycerides, mg/dl	215.7 ± 151.7	140.2 ± 64.3	<0.001
Apolipoprotein B, mg/dl	89.0 ± 24.1	63.3 ± 25.2	<0.001
OCT quantitative data			
Fibrous-cap thickness, μm	61.1 ± 18.5	159.1 ± 78.9	<0.001
Maximum lipid arc, °	241.2 ± 69.9	206.4 ± 75.3	<0.001
Mean lipid arc, °	174.8 ± 51.6	152.5 ± 55.7	<0.001
Lipid length, mm	9.9 ± 4.7	9.3 ± 4.9	0.010
Lipid index	1783.4 ± 1038.7	1476.0 ± 1042.7	<0.001
OCT qualitative data, *n* (%)			
TCFA	63 (64.9%)	13 (13.4%)	<0.001
Macrophage	71 (73.2%)	58 (59.8)	0.019
Micro-vessel	44 (45.4)	29 (29.9)	0.004
Cholesterol crystal	23 (23.7)	10 (10.3)	0.002
Thrombus	6 (6.2)	0	N/A
Spotty calcification	25 (26.0)	24 (24.7)	1.000
Disruption	9 (9.3)	0	N/A
CAG quantitative data			
Minimal lumen diameter, mm	2.0 ± 0.5	2.0 ± 0.5	0.824
Diameter stenosis, %	28.9 ± 10.5	29.5 ± 11.1	0.530
Lesion length, mm	11.3 ± 5.1	11.7 ± 5.0	0.069

Data are presented as mean ± SD or *n* (%). Quantitative data at baseline and at follow-up are compared using paired *t* test and qualitative data using McNemar test. *CAG* coronary Angiography, *LDL-C* low-density lipoprotein cholesterol, *HDL-C* high-density lipoprotein cholesterol, *OCT* optical coherence tomography, *TCFAs* thin-cap fibroatheromas; N/A not applicable. A *p*-value < 0.05 was considered statistically significant. ° arc of lipid content.

The lesion measurements done using CAG and OCT at baseline and follow-up are shown in Table 2. With statin therapy for 12 months, fibrous-cap thickness increased from 61.1 ± 18.5 μm to 159.1 ± 78.9 μm (*p* < 0.001); maximum lipid arc, mean lipid arc and lipid index significantly decreased (*p* < 0.001, all). In addition, we found that lesion length on CAG slightly increased (from 11.3 ± 5.1 mm baseline to 11.7 ± 5.0 mm follow-up, *p* = 0.069), but OCT showed that lipid length significantly decreased (from 9.9 ± 4.7 mm to 9.3 ± 4.9 mm, *p* = 0.01). Moreover, the OCT qualitative measurements showed that the prevalence of TCFA, macrophage infiltration, microvessels, and cholesterol crystals were significantly reduced at follow-up (*p* < 0.001, *p* = 0.048, *p* = 0.022, *p* = 0.031, *p* = 0.015, and *p* = 0.003, respectively), with the exception of the presence of calcification (*p* = 0.868). However, lumen dimensions as measured by CAG, including minimal lumen diameter and diameter stenosis (MLD: from baseline 2.0 ± 0.5 mm to follow-up 2.0 ± 0.5 mm, *p* = 0.824; DS: from 28.9 ± 10.5% to 29.5 ± 11.1, *p* = 0.53), were not significantly improved.

The correlations between the CAG and OCT findings of coronary lipid-rich plaques at baseline and also at follow-up were summarized in Table 3. The lesion longitudinal silhouette data showed that the whole length of plaque (lesion length) on CAG and the length of lipid pool on OCT were closely related at any single time points (*r* = 0.754, *p* < 0.001 at baseline; *r* = 0.639, *p* < 0.001 at follow-up); the lesion length also positively correlated with the lipid index. Moreover, diameter stenosis on CAG was significantly correlated with maximum lipid arc on OCT at baseline only (*r* = 0.209, *p* = 0.041). Minimal lumen diameter was not found significantly correlated with OCT measurements at any single time points.

**Table 3:** Correlations between the CAG and OCT data at single time points

CAG data	OCT data	Correlation	*p*-value
Baseline			
Minimal lumen diameter, mm	Fibrous-cap thickness, μm	−0.004	0.971
	Maximum lipid arc, °	0.062	0.545
	Mean lipid arc, °	0.071	0.494
	Lipid length, mm	0.134	0.197
	Lipid index	0.133	0.200
Diameter stenosis, %	Fibrous-cap thickness, μm	−0.169	0.099
	Maximum lipid arc, °	0.209	0.041
	Mean lipid arc, °	0.105	0.308
	Lipid length, mm	0.099	0.341
	Lipid index	0.115	0.265
Lesion length, mm	Fibrous-cap thickness, μm	−0.248	0.014
	Maximum lipid arc, °	−0.303	0.003
	Mean lipid arc, °	0.148	0.149
	Lipid length, mm	0.754	<0.001
	Lipid index	0.648	<0.001
Follow-up			
Minimal lumen diameter, mm	Fibrous-cap thickness, μm	−0.076	0.458
	Maximum lipid arc, °	0.086	0.410
	Mean lipid arc, °	0.087	0.433
	Lipid length, mm	0.104	0.334
	Lipid index	0.099	0.372
Diameter stenosis, %	Fibrous-cap thickness, μm	0.018	0.870
	Maximum lipid arc, °	−0.041	0.712
	Mean lipid arc, °	−0.014	0.902
	Lipid length, mm	0.072	0.506
	Lipid index	0.065	0.561
Lesion length, mm	Fibrous-cap thickness, μm	−0.112	0.300
	Maximum lipid arc, °	0.176	0.107
	Mean lipid arc, °	0.125	0.261
	Lipid length, mm	0.639	<0.001
	Lipid index	0.527	<0.001

Correlations between the CAG data and OCT data were analyzed by Pearson or Spearman ranks test. A *p*-value < 0.05 was considered statistically significant. ° arc of lipid content. ° arc of lipid content.

As shown in Table 4, the correlations between the changes in CAG and changes in OCT quantitative data from baseline to follow-up were analyzed. Change in minimal lumen diameter from baseline to follow-up was not significantly correlated with any changes in OCT quantitative data; neither was the change in diameter stenosis. And no significant correlations were found between change in lesion length on CAG and change in lipid length on OCT.

**Table 4:** Correlations between changes in CAG findings and changes in OCT measurements

Changes in CAG measurements	Changes in OCT measurements	Correlation	*p*-value
Change in minimal lumen diameter, mm	Change in fibrous-cap thickness, μm	−0.009	0.928
	Change in Maximum lipid arc, °	−0.096	0.357
	Change in Mean lipid arc, °	−0.079	0.440
	Change in Lipid length, mm	0.055	0.595
	Change in Lipid index	0.010	0.926
Change in diameter stenosis, %	Change in fibrous-cap thickness, μm	0.085	0.409
	Change in Maximum lipid arc, °	0.157	0.130
	Change in Mean lipid arc, °	0.059	0.568
	Change in Lipid length, mm	−0.053	0.605
	Change in Lipid index	−0.018	0.866
Change in lesion length, mm	Change in fibrous-cap thickness, μm	−0.119	0.247
	Change in Maximum lipid arc, °	−0.081	0.436
	Change in Mean lipid arc, °	−0.114	0.267
	Change in Lipid length, mm	0.092	0.371
	Change in Lipid index	−0.031	0.901

Correlations between changes in CAG findings and changes in OCT measurements were analyzed by Pearson or Spearman ranks test. A *p*-value < 0.05 was considered statistically significant

According to the FCT at baseline, plaques were classified into TCFAs (thin-cap fibroatheromas, fibrous-cap thickness < 65 μm) and ThCFAs (thick-cap fibroatheromas, fibrous-cap thickness ≥ 65 μm). Comparisons of lesion characteristics between TCFAs and ThCFAs were summarized in Table 5. Fibrous-cap thickness in both groups markedly increased (*p* < 0.001 both); and the maximum lipid arc of TCFAs, but not that of ThCFAs, significantly decreased over time. Diameter stenosis of TCFAs had a mean increase of 3.86% (*p* = 0.014) from the baseline to the 12-month follow-up; while DS of ThCFAs had a mean decrease of 1.14% (*p* = 0.26) over the same period. In addition, the mean lipid length significantly decreased over time (TCFAs: −1.1 mm, *p* = 0.005; ThCFAs: −0.40 mm, *p* = 0.218); however, the mean value of lesion length did not significantly change. Given the above, both TCFAs and ThCFAs showed significant improvements in lesion microstructures, but DS of ThCFAs grew narrower over time; although the lesion length of ThCFAs did not decreased at follow-up, the lipid length inside the lesion was significantly reduced. These results suggested that the lipid core decreased less and diameter stenosis increased in ThCFAs over time.

**Table 5:** Comparison of TCFAs and ThCFAs at baseline, at follow-up and changes over time

	TCFA (*n* = 63)	ThCFA (*n* = 34)	*p*-value
OCT measurements			
Fibrous-cap thickness, μm			
Baseline	49.8 ± 8.3	81.9 ± 13.4	<0.001
Follow-up	144.3 ± 80.9	186.4 ± 68.0	0.011
*p*-value	<0.001	<0.001	
Change	94.5 ± 79.8	104.5 ± 65.4	0.593
Maximum lipid arc, °			
Baseline	261.2 ± 66.6	204.7 ± 61.4	<0.001
Follow-up	219.4 ± 74.8	182.8 ± 71.3	0.022
*p*-value	<0.001	0.091	
Change	−41.8 ± 50.6	−22.0 ± 45.8	0.060
Mean lipid arc, °			
Baseline	190.8 ± 50.6	145.7 ± 39.7	<0.001
Follow-up	160.6 ± 56.9	137.6 ± 50.9	0.052
*p*-value	<0.001	<0.008	
Change	−30.3 ± 34.6	−8.1 ± 27.6	0.002
Lipid length, mm			
Baseline	10.7 ± 4.9	8.4 ± 4.0	0.019
Follow-up	10.4 ± 5.1	7.3 ± 3.7	0.003
*p*-value	0.218	0.005	
Change	−0.4 ± 2.3	−1.1 ± 2.0	0.142
Lipid index			
Baseline	2037.0 ± 1031.7	1083.6 ± 630.2	<0.001
Follow-up	1727.9 ± 1108.6	947.0 ± 705.9	<0.001
*p*-value	<0.001	0.007	
Change	309.1 ± 494.4	−136.6 ± 304.7	0.036
CAG measurements			
Minimal lumen diameter, mm			
Baseline	2.01 ± 0.58	1.99 ± 0.55	0.627
Follow-up	1.99 ± 0.55	1.98 ± 0.53	0.938
*p*-value	0.52	0.559	
Change	−0.02 ± 0.28	0.02 ± 0.24	0.686
Diameter stenosis, %			
Baseline	30.7 ± 10.2	23.8 ± 9.4	0.021
Follow-up	29.5 ± 10.9	27.7 ± 10.7	0.878
*p*-value	0.26	0.014	
Change	−1.14 ± 8.08	3.86 ± 8.55	0.008
Lesion length, mm			
Baseline	12.3 ± 5.6	9.37 ± 3.29	0.001
Follow-up	12.5 ± 5.4	9.98 ± 3.69	0.001
*p*-value	0.37	0.155	
Change	0.24 ± 1.88	0.61 ± 2.18	0.788

Data are presented as mean ± SD. Data at baseline and at follow-up are compared using paired *t* test; data between TCFAs and ThCFAs were compared by using unpaired *t* test. TCFAs, thin-cap fibroatheromas (FCT < 65 μm); ThCFAs, thick-cap fibroatheromas (FCT ≥ 65 μm). ° arc of lipid content.

**Fig. 1:**
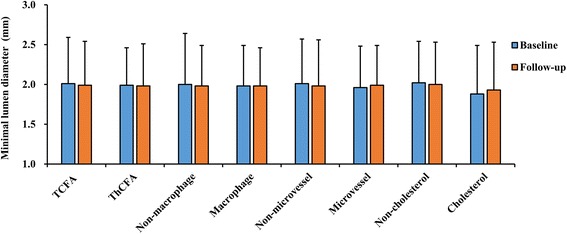
Comparisons of minimal lumen diameter in terms of OCT-derived plaque type. According to OCT-derived plaque types, plaques were classified into TCFAs and ThCFAs, with or without macrophage, or microvessels, or cholesterol crystals. Minimal lumen diameter showed no significant differences between groups

**Fig. 2:**
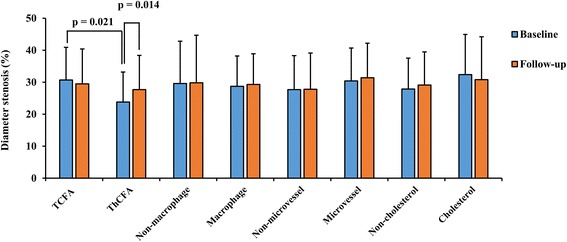
Comparisons of diameter stenosis in terms of OCT-derived plaque type. At baseline, diameter stenosis were significantly worse in TCFAs (*p* = 0.021). To compare the data at baseline and at follow-up in ThCFAs, we found that diameter stenosis was growing worse at follow-up (*p* = 0.014)

As shown in Figs. 1 and 2, lipid-rich plaques were also classified according to OCT-derived plaque types, such as the existence of a thin-cap < 65 μm, macrophage infiltration, microvessels, or cholesterol crystals. The differences between TCFAs and ThCFAs were described above. In addition, no significant differences of cross-sectional variables were observed on CAG measurements regardless of whether the lesions contained macrophage infiltration, microvessels, or cholesterol crystals.

## Discussion

To our knowledge, this is the first study to compare coronary lumen dimensions and plaque microstructural characteristics, and their progression during statin therapy using quantitative angiography and OCT imaging. The major results can be summarized as follows: 1) lipid length (OCT) was closely correlated with lesion length (CAG), but the other measurements on OCT and findings on CAG of non-culprit lipid-rich plaques were not significantly correlated; 2) although the microstructures were improved significantly at follow-up, the longitudinal and cross-sectional silhouette of the coronary lesions were not significantly improved; 3) compared with TCFAs, ThCFAs had a less decrease in the lipid index; ThCFAs had an increase in diameter stenosis at follow-up; 4) with 12-month statin therapy, no significant correlations between changes in CAG findings and changes in OCT measurements were observed.

It is generally known that the true architecture of the arterial wall and inside the atherosclerotic cannot be visualized directly using CAG, which is good at depicting the lumen as a longitudinal and cross-sectional silhouette [15, 23]. OCT, with its high-resolution histological images, has the capacity to delineate microstructural characteristics of atherosclerotic plaques, such as FCT, inflammation, and intra-plaque neovascularization [10, 13, 24]. However, so far, limited researches have been performed to explore correlations between CAG and OCT measurements on coronary atherosclerotic plaque. FCT and lipid burden are incontrovertible crucial factors for the rupture of lipid atherosclerotic plaque [12, 25]. The intravascular modality validated by OCT assessment and histological examination of the fibrous-cap thickness were highly consistent [26], while CAG had limitations to evaluate the microstructures and vulnerability of plaque [15, 24, 27]. Even so, in this study, we found a statistically significant but weak correlation between diameter stenosis by the standard method of evaluating lumen dimension with CAG and the maximum lipid arc with OCT at baseline but not at follow-up. However, if the follow-up duration of statin intervention is long enough, good correlations might be observed between OCT measurements and CAG findings on coronary plaques.

There were other important discoveries from this study. Lipid length seen on OCT results was strongly correlated with lesion length, but the correlation was weaker after 12 months of statin therapy. The weakened correlation might because statin therapy induces lipid core inside the lesion to shrink; however, lesion length was not significantly reduced at follow-up. In addition, the lipid index and microstructures of the lipid-rich plaques were markedly improved by follow-up time, but the luminal outline seen by CAG were not. These results are consistent with and extend those of previous studies documenting that CAG alone underestimates the composition of atherosclerosis and cannot evaluate the presence and magnitude of plaque microstructure progression [23, 24]. Intravascular OCT provides insights into plaque microstructures, which is needed in lipid-rich plaque progression/regression evaluation.

In the present study, we found that the changes in lumen dimension were not significantly correlated with changes in fibrous-cap thickness, neither with changes in lipid core content. In another analysis on evaluating coronary plaque progression/regression by OCT and IVUS, no significant correlations were found between changes in OCT parameters and changes in IVUS measurements [10].

The 12-month statin treatment has been reported to induce lipid-rich plaque stabilization, such as continuously thickening the fibrous cap and decreasing the presence of macrophage infiltration; however, the plaque volume determined by intravascular ultrasonography (IVUS) was not reduced [5], and the cross-sectional lumen architecture of coronary lesions were not significantly improved. This is most likely because statin induces fibrous-cap thickness of vulnerable plaques increasing, but the lipid core under the fibrous cap shrinking. Significant changes in atheroma volume and lumen dimensions cannot be discovered if follow-up time or the duration of statin treatment is too short. Results of SATURN trial using IVUS showed that maximal doses of statin-based lipid-lowering treatment for 104 weeks induced regression of atheroma volume [6]. Accordingly, the reduced vulnerability of lipid-rich plaques most likely occurs earlier than improvements in plaque volume and lumen dimensions during statin therapy; therefore, perennial statin-based lipid-modulating therapy and a long-term follow-up are necessary to attain the benefits of statins on decreased plaque volume and improved diameter stenosis.

OCT-derived TCFAs have been recognized as distinguishing features of so-called vulnerable or rupture-prone plaques [28]. Compared with TCFAs, ThCFAs had less of a decrease in the lipid index, but had an increase in diameter stenosis by follow-up period. The increase of fibrous-cap thickness in ThCFAs was not significantly different to that in TCFAs, while the decrease in lipid core burden of ThCFAs was much smaller than that of TCFAs. These findings might contribute to the differences between them in the changes in diameter stenosis, and lumen dimensions, which are most likely one process of statin-induced regression of ThCFAs; however, the present study was conducted for only 12 months. It remains unknown whether diameter stenosis of ThCFAs would improve with a longer-term follow-up.

### Limitations

First, it was inevitable that the retrospective study from a clinical registry introduced a potential selection bias. Second, because of the short duration and small size of population of our study, the changes in CAG data did not correlated with the changes in OCT measurements. Additional studies with larger populations and longer term follow-up might be needed. Third, the definition of OCT-derived TCFAs does not correspond exactly with the histological definition of TCFAs because OCT cannot measure the necrotic core size; the macrophage infiltration, micro-vessels, cholesterol crystals, thrombus, calcification, and disruption were not quantified in the present study. Finally, the general results of non-culprit, lipid-rich plaques in this study cannot be applied to other types of plaques.

## Conclusion

We found that the lipid length on OCT and the lesion length on CAG of lipid-rich plaques were closely correlated; however, the other microstructural characteristics measured by OCT and lumen dimensions by CAG were not significantly correlated. After 12-month statin treatment, we observed that microstructural characteristics measured were significantly improved, while CAG-derived lumen dimensions were not. Moreover, changes in OCT measurements were not significantly correlated with changes in CAG data induced by statin over time.

## Abbreviations

CAG: Coronary angiography
DS: Diameter stenosis
HDL-C: High-density lipoprotein cholesterol
LDL-C: Low-density lipoprotein cholesterol
MLD: Minimal lumen diameter
OCT: Optical coherence tomography
TCFAs: Thin-cap fibroatheromas
ThCFAs: Thick-cap fibroatheromas
